# Obstructive Sleep Apnea in Women: Specific Issues and Interventions

**DOI:** 10.1155/2016/1764837

**Published:** 2016-09-06

**Authors:** Alison Wimms, Holger Woehrle, Sahisha Ketheeswaran, Dinesh Ramanan, Jeffery Armitstead

**Affiliations:** ^1^ResMed Science Centre, Fraunhoferstraße 16, 82152 Planegg, Germany; ^2^University of Sydney, Fisher Road, Sydney, NSW, Australia; ^3^Sleep and Ventilation Center Blaubeuren, Lung Center Ulm, Ulm, Germany

## Abstract

Obstructive sleep apnea (OSA) has traditionally been seen as a male disease. However, the importance of OSA in women is increasingly being recognized, along with a number of significant gender-related differences in the symptoms, diagnosis, consequences, and treatment of OSA. Women tend to have less severe OSA than males, with a lower apnea-hypopnea index (AHI) and shorter apneas and hypopneas. Episodes of upper airway resistance that do not meet the criteria for apneas are more common in women. Prevalence rates are lower in women, and proportionally fewer women receive a correct diagnosis. Research has also documented sex differences in the upper airway, fat distribution, and respiratory stability in OSA. Hormones are implicated in some gender-related variations, with differences between men and women in the prevalence of OSA decreasing as age increases. The limited data available suggest that although the prevalence and severity of OSA may be lower in women than in men, the consequences of the disease are at least the same, if not worse for comparable degrees of severity. Few studies have investigated gender differences in the effects of OSA treatment. However, given the differences in physiology and presentation, it is possible that personalized therapy may provide more optimal care.

## 1. Introduction

Obstructive sleep apnea (OSA) is characterized by repetitive nocturnal complete collapses (apneas) or partial collapses (hypopneas) of the upper airway during sleep. These events are associated with oxygen desaturation and/or arousal from sleep. The severity of OSA is measured by the number of occurrences of airway collapse per hour (the apnea-hypopnea index [AHI]). OSA is the most common form of sleep-disordered breathing (SDB) and its prevalence has been increasing steadily, in part due to the global rise in obesity and in part due to changes to the recommended OSA scoring rules which were updated in 2012 to allow a broader definition of OSA [1].  Table 1 summarizes prevalence data for OSA in the general population.

OSA has been estimated to have a male-to-female ratio of between 3 : 1 and 5 : 1 in the general population and a much higher ratio of between 8 : 1 and 10 : 1 in some clinical groups [2–4]. Perhaps not surprisingly then, OSA has historically been regarded as a male disease [5]. Prevalence data do show that more men than women are affected by OSA; however, these differences are not reflected in clinical populations. This indicates that females are being diagnosed and treated for OSA less frequently than males.

## 2. OSA Classification, Diagnosis, and Symptoms

### 2.1. Sleepiness

It has been suggested that discrepancies between males and females in the prevalence of OSA could be a result of women frequently being misdiagnosed or underdiagnosed due to reporting different symptoms [4]. In the past, sleepiness has been seen as a key component of OSA. Obstructive sleep apnea syndrome (OSAS), which refers to OSA with accompanying symptoms, has been the main focus of treatment in the past. Furthermore, because the majority of clinical trial participants with OSA have been sleepy, it is still not clear whether asymptomatic OSA should be treated.

The Epworth Sleepiness Scale (ESS) is a tool used to measure the likelihood of falling asleep in certain situations and is commonly used to screen for OSA [5, 6]. Despite its widespread use, the ESS has not been validated for use in female OSA patients and has not been strongly associated with daytime sleepiness in female patients in population-based studies [5, 6]. In fact, even women who report similar levels of daytime sleepiness to men are less likely to have an ESS score >10 [6]. It is not clear why these differences occur; however, it is possible that women have a different threshold for feeling sleepy and/or complain differently about sleepiness compared with men [4].

### 2.2. Other Symptoms

Making a differential diagnosis of OSA in women might be more difficult given that they tend to present with more generalized daytime symptoms than men [4]. Women with OSA complain of symptoms such as insomnia, restless legs, depression, nightmares, palpitations, and hallucinations whereas men are more likely to report snoring and apneic episodes [7]. Women may consider their own snoring “unladylike” and therefore be less likely to mention it [4]. In addition, women are more likely to attend clinical appointments on their own, whereas men often attend with their partner [3]. Therefore, information from a partner on snoring and witnessed apneas may not be as readily available for women versus men. Less frequent reporting of “typical” OSA symptoms such as sleepiness and snoring by women, plus a higher prevalence of atypical symptoms such as insomnia, headache, anxiety, and depression, could contribute to the underevaluation of OSA in women, lower referral rates to sleep clinics, and underrepresentation in clinical studies [8, 9].

In a community-based sample, women with OSA reported the same symptoms as men across a range of severities, and snoring was the most significant predictor of OSA for both sexes [9]. However, a similar study of a population-based sample found that up to 40% of women with an AHI > 15/h did not report any of the classic OSA symptoms (snoring, witnessed apneas, and daytime sleepiness) [7].

### 2.3. Recognition and Diagnosis

It is clear that many women do report classic OSA symptoms, suggesting that factors other than symptoms also contribute to gender disparity in OSA populations [9]. These include failure of women to acknowledge OSA symptoms and seek medical help or failure of medical professionals to respond to OSA symptoms in women [4, 9]. Adding to the difficulty in correctly diagnosing female patients is the reporting of symptoms such as depression and anxiety, which are also more common in female than male patients without OSA [10].

Data from the Wisconsin University Sleep Laboratory showed that lower rates of recognition of OSA in women versus men only occurred in the subset of patients with an AHI of 5–20/h [11]. Their findings led the study authors to hypothesize that there may be greater gender-related differences in OSA symptom expression at lower AHI values, particularly with respect to characteristic symptoms such as snoring, witnessed apneas, and excessive daytime sleepiness.

Another difficulty in correctly diagnosing and treating OSA is understanding where the disease becomes significant and at what point treatment should be initiated. Large studies have typically shown an association between moderate-severe OSA and poor cardiovascular outcomes, whereas the same association has not been found in mild OSA [12]. Growing evidence suggests that mild OSA is associated with reduced quality of life, including general tiredness, fatigue, daytime impairment, difficulty concentrating and completing tasks, depressed mood, poor sleep quality and insomnia, and poor psychomotor performance [1, 13–17].

## 3. Gender Differences in the Upper Airway, Fat Distribution, and Respiratory Stability

Definitive explanations for differences between men and women in the symptoms, characteristics, and severity of OSA are not yet available, but various factors may contribute.

The focus of a number of studies has been on the upper airway. Magnetic resonance imaging has shown that airway length, the tongue, the soft palate, and the total amount of soft tissue in the throat are all smaller in women than in men [18]. Although, intuitively, a smaller airway might be expected to occlude more easily than a larger one, this does not seem to be the case. It appears that men have a longer, softer oropharynx and a larger, fatter, more posterior tongue, increasing the susceptibility of the large airway to collapse [4]. Upper airway collapsibility, determined by the pharyngeal critical closing pressure, has been shown to be less in women versus men when the severity of OSA is the same [19]. Sex differences in airway collapsibility were most evident during non-REM sleep, suggesting that men may be more susceptible to pharyngeal collapse than women during established sleep, but not during sleep transition [20].

Obesity is a well-recognized risk factor for OSA, and higher body mass index (BMI) is associated with greater severity of OSA for both sexes [18]. However, for the same AHI, women tend to be more obese than men [19, 27]. One potential explanation for this is differences in fat distribution between the sexes [28]. For the same BMI, men tend to have higher mean body weight, free fat mass, and neck circumference compared with women [29]. MRI studies have confirmed less pharyngeal fat and lower soft tissue volume in the neck for obese women versus obese men [30]. Upper airway fat distribution, particularly in the posterior tongue, appears to be important in the pathogenesis of OSA and is related to gender [4]. Upper body and visceral adiposity have been associated with reductions in lung function, including total lung capacity, forced vital capacity, and forced expiratory volume [31]. In addition, the independent effects of body fat distribution on lung function were more pronounced in men than in women [32].

Fat distribution might have physiological as well as mechanical effects in patients with OSA. Obese women, especially those with OSA, have been shown to have significantly increased hypercapnic and hypoxic responses, whereas this was not the case in obese men [33]. This adaptation might maintain adequate minute ventilation when the chest wall load is increased. In addition, men and women have been shown to require different levels of carbon dioxide in the blood to cause respiratory instability, and men were more susceptible to hypocapnic dysfunction during non-REM sleep than women. It is possible that women preserve ventilation output during hypocapnia more efficiently than men [34]. Indeed, the ventilatory response to hypercapnia has been shown to be greater in men than in women [35]. Thus, reduced lung function and decreased chemoresponsiveness are additional reasons why men are more susceptible to OSA than women.

There may also be gender differences in the arousal response to apneas. Jordan and colleagues found that during non-REM sleep men had a higher ventilatory response to apneas than women, but then they developed greater hypoventilation when they went back to sleep, especially in the supine position. This prolonged hypoventilation often leads to ventilatory instability upon returning to sleep. The study authors hypothesized that this may play a role in explaining why sleep apnea syndromes are more severe in men [36].

## 4. Manifestations

There are a number of gender differences in the manifestations of OSA; both the severity of OSA and its distribution across the sleep cycle differ in males and females. In patients with existing OSA, women had a significantly lower overall AHI compared with men (20.2/h versus 31.8/h; *p* < 0.001); AHI during non-REM sleep was also significantly lower in women versus men (14.6/h versus 29.6/h; *p* < 0.001) but there was no difference between females and males with respect to AHI during REM sleep (42.7/h versus 39.9/h, resp.), suggesting greater clustering of apneic events during REM sleep in women [37]. This study also showed that OSA in the supine position occurred almost exclusively in men, indicating that positional OSA is not really an issue for women [37]. Polysomnographic data from patients referred for suspected sleep disorders also showed that a difference between males and females in AHI was evident during stage 2 sleep, but not during REM sleep [38]. In addition, women had shorter apnea events and less severe oxygen desaturations than men (both *p* = 0.001) [38].

An interesting finding is that women are symptomatic at lower AHI cut-off values compared with men with the same AHI [9]. Females with an AHI of 2–5/h had a similar level of symptoms to men with an AHI of ≥15/h. In contrast, males with an AHI of 2–5/h were indistinguishable from those with an AHI of 0–2/h with respect to symptoms. One possibility is that the long-term effects of REM sleep disruption contribute to greater symptomatology at lower AHI values in women compared with men [39].

Another theory is that women may be more symptomatic because they have more episodes of upper airway resistance during sleep. Obstructive events can be thought of as a continuum from partial to complete upper airway obstruction. Upper airway resistance occurs early in this spectrum and describes events where resistance to airflow in the upper airway increases during sleep, presenting as flow limitation during polysomnography [40]. This increase in upper airway resistance could increase work of breathing, cause arousals and disrupted sleep, and impact daytime cognitive function [40]. Upper airway resistance alone, without complete obstructive apnea or respiratory disturbance, has been shown to produce clinical symptoms such as daytime fatigue and depression [41], both of which are symptoms reported by women with OSA.

Sleep architecture is another aspect that has been shown to differ between males and females. A study of 307 patients found that women took longer to fall asleep than men and, once asleep, had fewer awakenings and more slow wave (deep) sleep, despite no differences between the sexes in age, respiratory disturbance index, or oxygen saturation [42].

**Figure 1:**
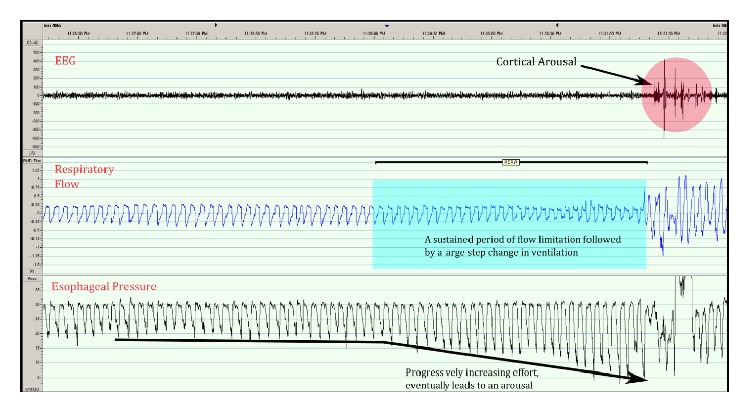
Respiratory effort-related arousals (RERAs). Trace shows a sustained period of flow limitation leading to increasing respiratory effort and arousal typical of RERAs. EEG: electroencephalography.

The occurrence of multiple episodes of upper airway resistance without frank apneas means that an AHI value may not provide a physician with a true indication of the degree of sleep fragmentation being experienced by patients. As a result, episodes during sleep where flow is reduced, respiratory effort increases, and the episode is terminated by an arousal have been termed respiratory effort-related arousals (RERAs) [40] (Figure 1). The importance of measuring and reporting RERAs has been emphasized by a task force of the American Academy of Sleep Medicine (AASM) [43].

Women with partial upper airway obstruction have been shown to have similar symptoms, including sleepiness, to women with OSA, resulting in a call for partial upper airway obstruction to be clinically recognized in the same way as OSA in women [44]. It has also been suggested that recognizing and understanding the different features of SDB in women are central for effectively detecting and treating the condition [45]. An update to the AASM scoring criteria in 2012 broadened the definition of OSA, and this may theoretically increase the number of patients with mild OSA. The AASM felt that there was sufficient evidence that hypopneas without associated oxygen desaturation, but rather hypopneas associated with arousal from sleep, were associated with significant daytime impairment and impacted quality of life to the point where treatment may be of benefit [1]. This is particularly relevant for female OSA patients because they are more likely to experience milder OSA with less severe oxygen desaturations. In addition, RERAs are now very rare with the new definition because most events of this nature can now be classified as hypopneas [1]. No prospective studies have investigated continuous positive airway pressure (CPAP) treatment in this newly defined group of patients with mild OSA; however, there is one randomized controlled trial underway which aims to do so (merge study, NCT02699463).

## 5. Menopause and Pregnancy

Differences between men and women in the prevalence of OSA decrease as age increases, largely as a result of a marked increase in the prevalence and severity of SDB in women after menopause [22, 46, 47]. Therefore, it has been suggested that female sex hormones have some sort of protective effect on upper airway patency and/or ventilatory drive [39]. The hormone progesterone is a known respiratory stimulant which increases chemoreceptor responses to hypercapnia and hypoxia and has been shown to increase upper airway muscle tone [48]. Progesterone levels decrease after menopause.

Hormones may also play a role in the distribution of body fat. Postmenopausal women have a higher fat mass compared to the period prior to menopause, and fat distribution is more likely to be in the upper body and trunk area compared with the lower body [49, 50]. In female volunteers, activity of the genioglossus muscle during wakefulness was lower in postmenopausal women compared with premenopausal women and significantly increased after 2 weeks of hormone replacement therapy [51].

Women may be at increased risk of OSA during pregnancy due to a number of factors. The growing uterus elevates the diaphragm, changing pulmonary mechanics [52]. In addition, during pregnancy, neck circumference increases [53, 54], nasal patency is reduced [55], and pharyngeal edema occurs [56]. Substantial increases in snoring, snorting/gasping, and witnessed apneas have been documented in pregnant women [54]. Snoring during pregnancy appears to be a risk factor for both pregnancy-induced hypertension and intrauterine growth retardation [57]. An ongoing study in this area will enrol 3702 women to understand the prevalence and outcomes of OSA during pregnancy [58]. Preliminary data from this group found that OSA affects 8.1% of pregnant women by the second trimester and that there was an association between OSA and hypertension and diabetes in this group [59].

There are limited data on the treatment outcomes of OSA during pregnancy, and no randomized controlled trials have been conducted in this area. Small studies have shown that CPAP treatment reduces blood pressure during pregnancy even when OSA is mild [60] and may improve pregnancy outcomes compared with untreated OSA [61, 62]; however, more research is required in this area.

## 6. Quality of Life

Several comparisons of women and men with untreated OSA have found that women report impaired quality of life. Women complain of more mood disturbances such as anxiety and depression, report low quality of life scores on a range of questionnaires, and display increased daytime fatigue, reduced sleep quality, and worsened neurobehavioral symptoms [63–66]. One limitation of these studies is that females were generally compared to males with OSA, rather than matched controls, meaning that there are no data on how female OSA patients differ from those in the general female population, where mood disturbances such as anxiety and depression can be common.

## 7. Health Consequences of OSA and Effects of Treatment 

OSAS has been associated with elevated cardiovascular risk and increased morbidity and mortality [67]. Observational studies have shown that adequate treatment of OSA with CPAP can reduce the incidence of cardiovascular events in patients with any severity of symptomatic OSA [68, 69]. The evidence for nonsleepy patients is mixed, with two short-term randomized studies showing no cardiovascular improvement in nonsleepy patients [70, 71]. However, a recent study by Barbé et al. included 725 nonsleepy patients with an AHI ≥20/h who were randomized to CPAP or a control group. There were fewer cases of new hypertension and cardiovascular events in the CPAP group, although this did not reach statistical significance (CPAP versus control group incidence density ratio (IDR) 0.81, confidence interval [CI] 0.61–1.06; *p* = 0.13). However, an analysis of those using PAP for ≥4 hours/night compared with the control group had an IDR of 0.69 (CI 0.50–0.94; *p* = 0.02) compared with an IDR of 1.12 (CI 0.77–1.64; *p* = 0.55) for those using CPAP <4 hours/night [72]. Due to the associations between OSA and harmful cardiovascular consequences, many researchers advocate for CPAP treatment of all patients, regardless of symptoms [73].

It has been postulated that nonsleepy patients will not be adherent to treatment; however, a recent large prospective trial has shown that long-term CPAP treatment is feasible in nonsleepy moderate-to-severe OSA patients [74].

### 7.1. Gender Differences in the Health Consequences of OSA

In the past, the belief that OSA was primarily a male disorder meant that clinical trial populations were comprised almost entirely of males [5]. Recently, studies have focused more specifically on the unique consequences of OSA in female patients.

Greenberg-Dotan et al. found that, compared to female controls, women with OSA were more likely to have a comorbid diagnosis including cardiovascular disease (odds ratio [OR] 1.4), hyperlipidemia (OR 1.5), diabetes (OR 1.6), asthma (OR 2.1), hypothyroidism (OR 1.6), arthropathy (OR 1.6), and reflux/gastritis (OR 2.5) [65].

Yaffe et al. studied a group of women with SDB and found that they were more likely to develop cognitive impairment or dementia than those without SDB. Cognitive issues were more likely to develop in patients with increased oxygen desaturation and higher periods of time spent in apnea or hypopnea [75]. Another study showed that female OSA patients experienced more brain white matter injury than their male counterparts [64]. It is hypothesized, though not yet known, that this change in white matter structure may be responsible for the worsened quality of life reported by women.

Sympathetically mediated responses to autonomic challenges in patients with OSA are blunted to a significantly greater extent in women versus men with OSA; this deficit is likely to reduce the effectiveness of BP regulation and brain perfusion [76]. In addition, it is possible that women with moderate sleep apnea are more susceptible to the adverse cardiovascular consequences of OSA than men, having been shown to have more marked endothelial dysfunction [77]. Certainly, untreated severe OSA has been independently and significantly associated with cardiovascular death in women [78, 79]. Conversely, the contribution of OSA to hypertension has been shown to be lower in women versus men [80].

The ability of CPAP treatment to improve outcomes in females has not been studied as extensively as in males. A prospective study by Campos-Rodriguez et al. evaluated the long-term outcomes of OSA in treated and nontreated female patients. They found that severe OSA was associated with increased cardiovascular mortality risk (adjusted hazard ratio 3.50, 95% CI 1.23–9.98) and that adequate CPAP treatment may reduce this risk [78].

In summary, the limited data available suggest that although the prevalence and severity of OSA may be lower in women than in men, the consequences of the disease are at least the same, if not worse [63].

## 8. OSA Treatment

In 2006, the American Academy of Sleep Medicine (AASM) reviewed all available evidence for CPAP and concluded that treatment was effective in improving quality of life in severe and moderate OSA, but there was insufficient evidence for the effectiveness of CPAP in mild OSA [81]. More recent data showed that CPAP treatment significantly improved quality of life compared with sham treatment in 223 mild-moderate patients (AHI 5–30/h) [82]. In addition, CPAP treatment was associated with significant improvements in quality of life in female OSA patients on a number of measures including daytime functioning, activity levels, daytime sleepiness, mood disturbances, and impact of sickness on daily life [63]. Campos-Rodriguez et al. recently published the first study to review the quality of life impact of CPAP treatment in women with moderate-to-severe OSA. Compared with the control group, the CPAP group had significantly greater improvements in all quality of life measures, including sleepiness (*p* < 0.001), mood (*p* = 0.012), anxiety (*p* = 0.014), and depression (*p* = 0.016) [83].

Craig et al. randomized 391 nonsleepy mild OSA patients to CPAP therapy or standard care for 6 months and found that CPAP improved daytime sleepiness (based on ESS scores), objective sleepiness, and self-assessed health status (SF36), but not vascular health risk [84]. Interestingly, Craig et al. found no relationship between OSA severity and improved quality of life, indicating that the severity of OSA may not accurately predict CPAP effectiveness. In 2016, the American Thoracic Society again reviewed the evidence available for CPAP treatment of mild OSA. They concluded that patients with sleepiness may benefit from treatment and that CPAP may also improve quality of life. They found that there was still insufficient evidence to understand the impact of mild OSA treatment on cardiovascular events, stroke, and arrhythmias [17].

### 8.1. Gender Differences in OSA Treatment

Sex differences in the response to different OSA treatment strategies have not been extensively studied to date. The limited data available indicate that usage is similar between males and females. A review of a database of 4281 patients found that average daily CPAP usage in males patients was slightly higher than in female patients; however, average usage time in both genders was high (377 ± 94 versus 370 ± 96 min) [85]. A similar analysis followed up a group of 708 women for a median of 6.2 (4.2–7.7) years. Overall long-term compliance with treatment was good in female patients, with median daily usage of 6 hours per day (interquartile range 4–7); 82.8% of patients were still using CPAP after 5 years, and 79.9% were still on CPAP at 10 years [86].

Clinical trials have indicated that males require higher CPAP pressure than females, after adjusting for baseline OSA severity and BMI [87, 88]. However, there do not appear to be differences between men and women in the types of interfaces used for CPAP or overall satisfaction with mask treatment [89].

Given that there are marked differences between men and women in the physiology and presentation of OSA, it is possible that treatment options specifically targeting female presentations of OSA may result in better treatment outcomes for these patients [85, 87, 88]. One recent bench test has found that there are significant differences in the way commercially available CPAP devices respond to flow limitation common in female patients [90]. Personalized medicine has not made major inroads into OSA, despite the potentially different gender and potential symptom specific phenotypes [91]. One commercially available CPAP device contains an algorithm which aims to address female-specific OSA characteristics. This device was tested in a randomized, double-blind, crossover clinical trial and was found to be as effective as standard CPAP, with a significant reduction in residual flow limitation and lower mean pressures [92]. An ongoing clinical study is investigating the use of this device on quality of life in women, with outcome measures including daily functioning, sleepiness, depression, sexual function, and sleep quality (NCT02400073).

Non-CPAP treatments have rarely been studied for gender specific effects. Mild patients are often instructed to lose weight; however, this may be more beneficial for males than females based on the fat distribution in the upper airway of males [93].

Mandibular Advancement Devices (MADs) are a treatment option for those with mild-moderate OSA or those who have rejected CPAP. One large study found that female gender was a predictor of MAD treatment success, particularly when OSA was mild [94]. However, more research is needed in this area.

## 9. Conclusion

A growing body of evidence suggests that there are substantial differences between females and males in the symptoms, diagnosis, and consequences of OSA. The majority of existing data relate to populations with a predominance of males, particularly with respect to treatment. Better knowledge of gender differences in OSA will help to improve the awareness and diagnosis of OSA in women, and the development and availability of therapeutic options that take into account differences in the physiology and presentation of OSA in women could have the potential to improve outcomes for these patients.

## Figures and Tables

**Table 1:** Estimated population prevalence of OSA.

Study	Mild OSA (AHI ≥ 5/h)		Moderate-to-severe OSA (AHI ≥ 15/h)	
	Males	Females	Males	Females
Young et al. [2]	24%	9%	9%	4%
Redline et al. [21]^*∗*^	—	—	26%	13%
Bixler et al. [22, 23]	17%	—	7%	2%
Durán et al. [24]	26.2%	28%	14%	7%
Peppard et al. [25]	—	—	13.5%	6%
Franklin et al. [5]^∧#^	—	50%	—	26%
Heinzer et al. [26]^#^	34%	38%	49.7%	23.4%

^*∗*^Respiratory disturbance index (RDI) rather than AHI given.

^∧^Women aged 20–70 years.

^#^Updated scoring criteria (AASM 2012) used.
